# Seroprevalence of herpes simplex virus type 1 among people living with HIV in Mbeya, Tanzania

**DOI:** 10.1186/s12879-020-05301-2

**Published:** 2020-08-05

**Authors:** Habakkuk Mwakyula Issakwisa, Gloria Reginald Mbwile, Godlove Fred Mbwanji, David Daniel Nassoro, Nyanda Elias Ntinginya, Anthony Ambikile Nsojo

**Affiliations:** 1 Mbeya Zonal Referral Hospital, Mbeya, Tanzania; 2 University of Dar es Salaam Mbeya College of Health and Allied Sciences, Mbeya, Tanzania; 3 Mbeya Regional Referral Hospital, Mbeya, Tanzania; 4 NIMR-Mbeya Medical Research Center, Mbeya, Tanzania

**Keywords:** Prevalence, HSV-1, Human Immunodeficiency Virus, Mwakyula

## Abstract

**Background:**

Despite the significant decline in the prevalence of HIV in Tanzania, the prevalence rates in Mbeya, Iringa, and Njombe regions are higher than the national average and have remained stable for years. The current stable HIV prevalence may be driven by factors such as a high incidence of sexually transmitted infections (STIs) and high-risk behaviours. In sub-Saharan Africa, it has previously been observed that up to 50% of HIV cases were attributed to herpes simplex type 2 (HSV-2) among low-risk populations. Because the proportion of sexually transmitted HSV-1 is rising, it is essential to study the interaction between HSV-1 and HIV infections.

**Methods:**

We conducted a study in Mbeya region using the archived blood sera of participants from the recently completed EU-funded EMINI project. A specially designed questionnaire was used to obtain the social and demographic characteristics of the study participants in the database. We tested archived participants’ sera for herpes simplex virus type 1 using Virotech HSV-1 (gG1) IgG ELISA (Enzygnost, Behring, Germany). Univariate and multivariate Poisson regression models were used to identify factors associated with HSV-1.

**Results:**

A total of 640 adults were randomly recruited after stratification by HIV status (318 were HIV positive), age, and sex. The overall seroprevalence of HSV-1 in the study population was 92.1%. The extrapolated seroprevalence estimate of herpes simplex virus type 1 in the general population was 95.0% (96.0% in males versus 94.0% in females). Males and females were equally affected by HSV-1. HSV-1 was less prevalent in HIV-positive individuals than in HIV-negative individuals.

**Conclusion:**

People living with HIV were less likely to be HSV-1 seropositive. Further prospective studies are necessary to conclude a causal association.

## Background

In mainland Tanzania, the prevalence of human immunodeficiency virus (HIV) infection among adults aged 15–49 years is 4.7%, and the prevalence among females is twice that among males (6.2% vs. 3.1%). Although there is evidence of a significant decline in the prevalence of HIV in Tanzania, the prevalence rates in Mbeya, Iringa, and Njombe regions (9.3, 11.3, and 11.4% respectively) are higher than the national average and are stable [1].

The current stable HIV prevalence may be driven by a high incidence of sexually transmitted infections (STIs) and high-risk behaviours among patients in sub-Saharan Africa who are taking antiretroviral therapy (ART) [2, 3]. In contrast, individuals undergoing ART in northern Africa tend to adopt protective behaviours more frequently than those who are not undergoing treatment [4].

Herpes simplex virus type 1 (HSV-1) predominantly causes orolabial ulcers and is acquired during childhood, while HSV-2 causes genital herpes that is commonly associated with sexual activities. Sufficient evidence has demonstrated an increase in HSV-1 anogenital isolates [5–10].

There is some evidence that a large proportion of HIV infections is attributable to HSV-2 [11, 12]. In sub-Saharan Africa, it has previously been observed that among low-risk populations, 25% [13] to 50% [14] of HIV-1 cases were attributable to HSV-2. HSV-2 disrupts the epithelial barrier and recruits immune cells, which are targets for HIV in the genital area, thereby facilitating the entry of HIV [15].

Since the proportion of sexually transmitted HSV-1 is rising and HSV-1 leads to the persistent shedding of oropharyngeal mucosal ulcers and ulcerations of genital mucosa and skin [16], it is essential to study the potential role of HSV-1 as a risk factor for HIV infection.

## Methods

The current study was a population-based cross-sectional study that analysed data and blood from a subset of participants who were recruited into the EMINI (Establishment of the Infrastructure to Evaluate and Monitor the Impact of New Interventions) cohort.

EMINI was a population-based cohort study that was conducted in Mbeya region between May 2005 and 2009. It included 20,000 people enrolled from 10% of all households in nine randomly selected communities (urban, semi-urban, and rural). The overall objective of the EMINI project was to contribute to the general improvement of health by controlling communicable diseases in Mbeya region.

During each of the annual study visits, a research team visited every participant to conduct a general physical examination, perform interviews (particularly about sexual risk behaviours), and collect several specimens, including blood samples.

The blood specimens collected from the participants were then tested for HIV-1 in the NIMR-MMRC central laboratory at the Mbeya Zonal Referral Hospital in accordance with the existing national HIV testing algorithm. During each survey, serum from every participant was stored at − 80 °C for future use.

The HSV-1-specific gG1 IgG assay (Enzygnost, Behring, Marburg, Germany) was used to examine samples for HSV-1. This assay is an ELISA for the semiquantitative and qualitative detection of IgG antibodies against HSV-1 in human serum. Standard operating procedures and data worksheets for this kit were made and followed in accordance with the manufacturer’s recommendations.

We linked the assay results to each participant’s demographic characteristics and their responses to the sexual risk behaviour checklist. Data regarding age, sex, sociodemographic status, behaviours of participants, and physical characteristics were all collected during interviews in the participants’ households using PDAs. The data were downloaded into Microsoft Access databases and imported into Stata 16.0 (StataCorp, College Station, Texas 77,845 USA) statistical software, which was used for data analyses.

Overall, 7287 participants in the EMINI cohort were between the age of 15 and 49 years old. Participants meeting the inclusion criteria were randomized using Stata 16 after being stratified by age, sex, and HIV status. Stratification was necessary because otherwise, the number of HIV-positive participants would have been too low for meaningful comparisons.

Each participant was assigned a random number, and 23 participants were initially selected per stratum (overall = 23 participants × 28 strata = 644 participants). A stratum (plural strata) refers to a subset of the population that is being sampled. Strata with fewer than 23 participants were filled from higher age strata with the same sex and HIV combination. For each stratum, 10 participants were also selected for replacement in case the selected initial samples could not be found (total number of replacements was 280). Responses from EMINI interviews were used to assess the socio-demographic characteristics of participants.

We performed all analyses in Stata. Univariate and multivariate associations of different factors with HSV-1 infection were analysed using Poisson regression with robust variance estimates.

## Results

We randomly selected a total of 640 subjects from nine wards of the Mbeya region, and the sample has an equal distribution of sex and HIV status. The majority of the study population had completed a primary level of education (58.4%), were farmers (70.0%), were involved in seasonal activities (50.5%), were married with one spouse (55.0%), and were Christians (77.5%). Some of the socio-demographic characteristics of these participants are shown in Table 1.

**Table 1:** Frequency distribution of sociodemographic characteristics (*n* = 640)

Demography	Frequency	Percent
HIV status		
Negative	322	50.3
Positive	318	49.7
Sex		
Female	319	49.8
Male	321	50.2
Age group		
15–19	77	12.0
20–24	83	13.0
25–29	106	16.5
30–34	101	15.8
35–39	90	14.1
40–44	90	14.1
45–49	93	14.5
Level of education		
No formal education	88	26.9
Completed primary	374	58.4
After primary	94	14.7
Marital status		
Never married	154	24.1
Married-one spouse	352	55.0
Others	134	20.9
Religion		
No religion	107	16.7
Christian	496	77.5
Others	36	5.8

Six hundred and twenty-nine sera were tested for HSV-1; 18 sera were indeterminate, and in 11 participants, the volume of sera was insufficient to run the test. None of the sera with indeterminate results were retested. From 611 participant sera tested for herpes simplex virus type 1, 51.2% were males, and 52.4% of sera came from HIV-negative individuals.

The overall seroprevalence of HSV-1 infection was 92.1%, with males and females having a seroprevalence of 94.2 and 89.9%, respectively. A total of 95.7% of HIV-negative and 83.2% of HIV-positive females were reactive to herpes simplex virus type 1 antibodies. These proportions were slightly lower than the seroprevalence of 96.9 and 91.6% among HIV-negative and HIV-positive males, respectively.

We stratified the study participants by HIV status, sex, and age to have enough participants in each stratum. Approximately 50% of study subjects were infected with HIV; the prevalence of HIV infection among the general population of Mbeya was 9.3%. The unadjusted seroprevalence did not interfere with the analysis of the seroprevalence of HSV infections in HIV-infected and uninfected individuals; however, the unadjusted data did not represent the overall seroprevalence in the general population. After extrapolating the current results to the age, sex, and HIV status distribution of the EMINI study population, the seroprevalence estimate of herpes simplex virus type 1 in the general population was 95.0% (96.0% in males versus 94.0% in females).

A chi-square test of independence was performed to examine the relationship between sex and HSV-1 seropositivity. The relation between these variables was significant, X^2^ (1, *N* = 611) = 3.929, *p* = .047461. Men were more likely than women to be HSV-1 positive (Table 2).

**Table 2:** Prevalence of herpes simplex virus type 1 by sex and HIV status

	Male (Expected total) [X^2^]	Female (Expected total) [X^2^]	Total
HSV-1			
Positive	295 (288.41) [0.15]	268 (274.59) [0.16]	563
Negative	18 (24.59) [1.77]	30 (23.41) [1.85]	48
	313	298	611
	HIV Positive	HIV Negative	
Positive	255 (268.14) [0.64]	308 (294.86) [0.59]	563
Negative	36 (22.86) [7.55]	12 (25.14) [6.87]	48
	291	320	611

The chi-square statistic is 3.929. The *p*-value is .047461. This result is significant at *p* < .05

The correlation between HSV-1 and HIV status was tested using chi-square and Z score tests. A chi-square test of independence showed that there was a significant association between HSV-1 seropositivity and HIV status, X^2^ (1, *N* = 611) = 15.6482, *p* = .000076; these findings were consistent with additional analyses, with a Z score of − 3.9558 and a *p*-value of .00004. The result suggests that either disease, namely, HIV, or HSV-1, would be protective against the other.

Poisson regression analysis with robust variance estimates showed that there was no significant change in the trend of seroprevalence of herpes simplex virus type 1 over age by HIV status.

To assess the univariate association of sex and HIV status with HSV-1 infection, Poisson regression analysis with robust variance estimates for stratified variables was used (Table 3). There was no significant difference in HSV infections between females and males. People with HIV were less likely to have HSV-1 infections. When we included the same variables in a multivariate model, the results revealed a 7% decrease in the seroprevalence of HSV-1 among HIV-positive patients.

**Table 3:** Univariate and multivariate association of various factors with HSV-1 infection

Variable	Stratum	Number in stratum	Prevalence ratio	95% CI	*P*-value
Univariate					
Sex					
	Female*	298	1	–	–
	Male	313	1.05	(1.00 to 1.10)	0.050
HIV status					
	Negative*	320	1	–	–
	Positive	291	0.91	(0.87 to 0.96)	< 0.001
Multivariate					
Sex					
	Female*	298	1	–	–
	Male	313	1.04	(0.99 to 1.10)	0.110
HIV status					
	Negative*	320	1	–	–
	Positive	291	0.93	(0.89 to 0.98)	0.004

Results of Poisson regression with robust variance estimates for stratified variables. Univariate and multivariate models for sex, age, and HIV status, all other variables adjusted for sex, age, and HIV status

## Discussion

This study presents the first report of the seroprevalence of herpes simplex virus type 1 in the general population of Tanzania based on HIV status. The unadjusted seroprevalence of herpes simplex virus type 1 infections in this study population was 92.1%, and the seroprevalence estimate for the general population was 95.0%. The rate in our study is higher than the prevalence rates in the general population of the following countries: the United States of America, 67% [17]; Israel, 59.8% [18]; and Switzerland, 80% [19]. The estimate from our study is, however, comparable to the general populations of some regions in Syria [20], rural Uganda (91.2%) [21], and the urban region of Tanzania (92%) [22].

In contrast to other studies where the seroprevalence of HSV-1 was significantly higher in females [23–25], this study shows that men are more often infected than women. However, this association was no longer significant after adjusting for other factors in the multivariate model (PR 1.04, *p*-value = .110).

Surprisingly, the present study shows a lower seroprevalence of herpes simplex virus type 1 infection among HIV-positive individuals compared to HIV-negative individuals (87.6% vs. 96.3%). In univariate and multivariate analyses, HIV positivity was associated with lower HSV-1 seropositivity.

This is the first study to report the low seroprevalence of HSV-1 in HIV-infected individuals. These findings were derived from a seroprevalence study, and henceforth the presence of genital herpes by HSV-1 was not assessed. The finding could also have been confounded by other dynamics such as age at sexual debut, behavioral factors like condom use and herpes simplex type 2 infections; and therefore, this finding in this study design should, consequently, be interpreted with caution.

Do HIV positive individuals receive more information about condom use than non-positive, and henceforth they have a better chance of protection against sexually transmitted infections, such as HSV-1? Some studies in sub-Saharan Africa have already described low condom use among individuals on antiretroviral drugs [2, 3]. While most HSV-1 infections are acquired in childhood but changes in HSV-1 epidemiology have been witnessed showing acquisition in later age [26]. Another study has also reported that HSV-1 infection is not transmitted sexually in HIV-infected individuals [27]. Meanwhile, from our research group, in the same participants’ sera, the seroprevalence of HSV-2 IgG was higher in HIV-positive individuals (unpublished observation).

There is a paucity of epidemiological studies that describe the interaction of HSV-1 and HIV. The present findings support the observed negative in vitro interaction between HSV-1 and HIV. Calistri et al. [28] used the CD4 human lymphoblastoid cell line CEM that was infected chronically by HSV-1, CEM_HSV_, and superinfected the cells with HIV-1. They found that HIV-1 growth was inhibited, probably due to HSV-1 failing to induce HIV-1 gene expression through the transactivation of the long terminal repeat (LTR).

Correspondingly, one more study has found that membrane-bound HSV-1 glycoprotein D is required for inhibiting the release of infectious HIV-1 [29]. Likewise, Polpitiya et al. found HSV-1 proteins, such as glycoprotein M, US3, and UL24, potently restricted the replication of HIV-1 and also prevented the incorporation of the HIV-1 gp120/gp41 into virus particles [30].

## Conclusions

The seroprevalence of HSV-1 in this population was high. People living with HIV were less likely to be HSV-1 positive. The current study design is susceptible to many confounding factors, and it is difficult to determine the causal association. Further prospective studies are necessary to conclude a causal relationship.

## Data Availability

The datasets generated during and/or analysed during the current study are available from the corresponding author upon reasonable request.

## Abbreviations

HIV: Human Immunodeficiency Virus
AIDS: Acquired Immune Deficiency Syndrome
HSV-1: Herpes Simplex Virus Type 1
EU: European Union
EMINI: Establishment of the Infrastructure to Evaluate and Monitor the Impact of New Interventions
ELISA: Enzyme-Linked Immunosorbent Assay
gG1: Glycoprotein G 1
NIMR: National Institute for Medical Research
MMRC: Mbeya Medical Research Center
PDAs: Personal Digital Assistants
TX: Texas
MMReC: Mbeya Medical Research and Ethics Committee
CI: Confidence Interval
CEM: Contagious Equine Metritis
